# Valorization of Ornamental Stone Processing Waste for the Synthesis of LTA- and SOD-Type Zeolites

**DOI:** 10.1021/acsomega.5c10482

**Published:** 2026-02-26

**Authors:** Eliomar P. Céleri, Carmem C. M. da Silva, Damaris Guimarães, Valdemar Lacerda, Audrei G. Barañano

**Affiliations:** † Núcleo de Competências Em Química Do Petróleo, 680695 Universidade Federal Do Espírito Santo, Av. Fernando Ferrari, 514, Vitória, Espírito Santo 29075-910, Brazil; ‡ Departamento de Engenharia Rural, Universidade Federal Do Espírito Santo, Alto Universitário, Alegre, Espírito Santo 29500-000, Brazil

## Abstract

The conversion of ornamental stone waste, rich in quartz and potassium feldspar, into precursors for LTA and SOD zeolite synthesis was investigated via alkaline activation at 850 °C, followed by hydrothermal crystallization without autoclaves. This approach promoted complete amorphization of the material, facilitating the breakdown of the original crystalline framework and the incorporation of sodium ions, which are essential for structural rearrangement during zeolite formation. LTA zeolite was obtained after 6 h of crystallization, reaching 99.94% crystallinity, and progressively transformed into the SOD phase over 48 h, achieving 87.56% crystallinity. This phase transition was closely related to variations in the Na_2_O/Al_2_O_3_ molar ratio and the conditions governing nucleation and crystal growth. Solid-state ^29^Si NMR analyses revealed local rearrangements in the aluminosilicate framework during the LTA → SOD transformation, highlighting the key role of Na^+^ redistribution in structural evolution. Complementary ^27^Al NMR confirmed the exclusive presence of tetrahedrally coordinated aluminum and the absence of Brønsted acid sites, indicating a basic character of the synthesized zeolites. These results demonstrate the technical and environmental feasibility of using ornamental stone waste as a raw material, providing a sustainable and economically competitive route to produce functional zeolites with potential applications in catalysis, adsorption, sensing, and gas separation technologies.

## Graphical abstract

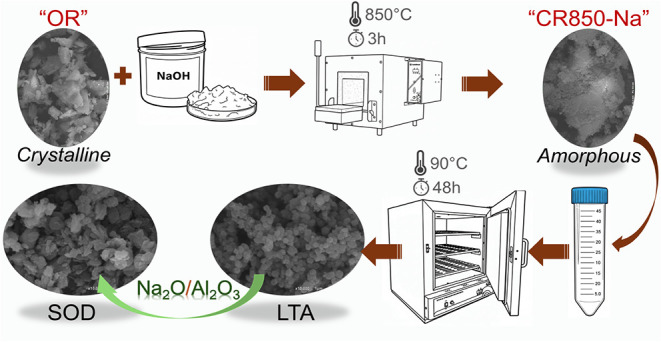

## Introduction

The ornamental stone industry in Brazil is one of the most significant sectors of the national mining industry, standing out for its economic and social relevance. The country holds a leading global position in the production and export of ornamental stones, particularly granite and marble. Between January and November 2025, the Brazilian ornamental stone export market reached 1.14 billion U.S. dollars and 1.87 million tons, highlighting the sector’s importance to the national economy.^1^

Ornamental stones, widely used in flooring, cladding, and countertops, can be primarily classified as granite, marble, and quartzite. Granite is composed of quartz, feldspar, and iron-bearing minerals; marble consists mainly of calcite and dolomite; while quartzite is essentially composed of quartz.^2^ During the processing of these stones, from quarry extraction to industrial cutting and polishing, solid and slurry wastes are generated due to the use of water for tool cooling and slab polishing, in addition to particulate residues resulting from sawing with diamond wire saws.^3,4^

It is estimated that about 30% to 40% of the volume extracted from quarries is converted into waste, totaling between 690,000 and 920,000 tons per year.^5^ These residues contain metal contents such as Fe, Mg, Ca, Cu, Cr, Pb, and Cd, whose presence may alter the local ecosystem. Additionally, particles with sizes between 1 and 60 μm can block soil pores, compromising its permeability and fertility.^6^ Such environmental impacts may negatively affect human health and biodiversity.^7^

Sludge wastes from ornamental stone processing, which are typically disposed of in landfills, can be effectively used as secondary raw materials to replace conventional aggregates (siliceous fillers) in self-compacting mortars, without increasing costs and contributing to the reduction of dependence on natural raw materials.^8^ Azevedo et al.^9^ reported that replacing conventional lime in the cement industry with industrial solid wastes, including those derived from ornamental rocks, could generate annual savings of approximately 250 million U.S. dollars for the sector. Other applications of these wastes include the manufacture of red-fired coatings,^5^ the production of soda-lime glass,^10^ the preparation of mortars,^6^ interlocking paving blocks,^11^ belitic cement,^4^ their incorporation into clay ceramics,^12^ the development of sustainable clay-based cosmetic mask formulations,^13^ and their use as sand substitutes in concrete, assisted by silane coupling agents.^14^

An alternative for the utilization of ornamental rock waste, particularly granite, is the synthesis of zeolites, as these wastes are rich in aluminosilicates, which are key precursors for the formation of the material. Zeolites possess a microporous crystalline structure composed of aluminum and silicon oxide tetrahedra that share oxygen atoms at the vertices, creating interconnected polyhedra that form a three-dimensional network with porous channels ranging from 0.2 to 1.2 nm, occupied by cationic ions, mainly alkali and alkaline earth metals, which neutralize the negative charge of tetrahedral aluminum.^15,16^ Synthetic zeolites allow the adjustment of chemical properties and the number of active sites, being preferred for specific applications, whereas natural zeolites are more suitable as molecular sieves.^17^ These materials are widely used in the adsorption of metal ions,^18,19^ proteins^20^ and dyes,^21^ enzymatic immobilization,^22^ catalytic pyrolysis,^23^ antitumor remediation^24^ and biofuel synthesis.^25^

Zeolite synthesis can be carried out using either pure chemical reagents or industrial wastes rich in silicon and aluminum oxides, providing economic and environmental benefits by promoting waste valorization and reducing the extraction of natural resources.^26^ Various types of zeolites have already been synthesized from silicoaluminous wastes, including Linde Type A (LTA) zeolites derived from glass and aluminum wastes^27^ coal fly ash,^28^ lithium leaching residues^29^ and rice husk combined with diatomite;^24^ GIS-type zeolites obtained from municipal solid waste incineration fly ash^30^ and kaolinite-rich construction and demolition wastes;^31^ and faujasite (FAU) zeolites synthesized from acid-treated waste glass powder^32^ and rice husk ash.^33^

The hydrothermal method, also referred to as the solvothermal method, is the most extensively studied for zeolite synthesis. It involves the addition of aluminum and silicon precursors to an alkaline solution (pH > 8.5), followed by transfer to autoclaves at temperatures between 80 and 350 °C, promoting the growth of highly crystalline and single-phase crystals.^34^ However, the use of mineral wastes in this method presents limitations when they contain high amounts of thermally stable minerals, such as feldspars and quartz. An effective alternative is alkaline activation with NaOH, a procedure that promotes the breakdown of crystalline structures and the formation of soluble silicates in solution. This process increases the availability of silica and alumina in a reactively accessible form, which is crucial for the subsequent formation of zeolites during the hydrothermal step.^35^

Among the benefits of alkaline activation are the improved dissolution of active components, reduced crystallization time, and lower consumption of additional reagents. However, alkaline activation also presents disadvantages, such as high energy consumption, since the activation temperature generally ranges between 600 and 1000 °C for 1.5 to 4 h.^36^

In this study, ornamental rock wastes rich in quartz and potassium feldspar were used as precursors for zeolite synthesis, employing the hydrothermal method without autoclave assistance, following a pretreatment via alkaline activation. This approach allows the valorization of abundant waste from the ornamental rock industry, converting low-value materials into functional zeolites, while simultaneously overcoming limitations associated with the presence of thermally stable minerals.

## Methodology

### Treatment of Ornamental Rock Waste

The ornamental rock waste was obtained as a donation from an industry located in the municipality of Cachoeiro de Itapemirim, Espírito Santo, Brazil. This waste, composed of slabs with approximate dimensions of 45 × 30 × 5 cm, underwent a crushing process followed by grinding in a ring mill to produce a powdered form of the waste. The resulting powder was passed through a 75-μm mesh sieve, and the finest fraction was collected, stored, and designated as “OR.”

To obtain reactive aluminosilicates necessary for zeolite synthesis, the alkaline activation method described by Kong and Jiang^37^ was adopted with adaptations. In this procedure, the OR powder was mixed with solid NaOH in a mass ratio of 1:1.5 to break down the crystalline structure of OR and was calcined at 850 °C for 3 h, then cooled inside the furnace to 100 °C, after which it was removed and stored in a vacuum desiccator. The calcined material was dispersed in distilled water at a ratio of 1 g per 20 mL. The solid suspended in this solution was separated by filtration, washed with distilled water until reaching pH 9, and subjected to a drying process at 105 °C for 12 h.

The obtained solid was designated as “CR850-Na,” with 850 representing the calcination temperature. To evaluate the leaching of Si and Al species into the aqueous phase during the dissolution step of the calcined material, an aliquot of the aqueous phase was collected and designated as “LS-CM.” To assess the necessity of alkaline activation for obtaining amorphous material, the OR powder was also calcined under the same conditions as CR850-Na, but without the addition of NaOH. This sample was named “CR850.”

### Zeolite Synthesis

For zeolite synthesis, adaptations were made from the works of Aliasmaeel et al.,^38^ Su; Ma; Chuan^39^ and Huo et al.^40^ The CR850-Na precursor was homogenized at a ratio of 1 g per 10 mL of a 14 wt % NaOH solution. This mixture was stirred using a magnetic stirrer for 12 h at room temperature until a gel was formed, which was then subjected to a crystallization process in 50 mL autoclavable polyethylene bottles. Crystallization was carried out over 48 h at a constant temperature of 90 °C. During this period, samples were withdrawn after 6, 12, 24, and 48 h, and were designated as “Z-X,” where X represents the time at which the sample was collected.

### Characterizations

Thermogravimetric analysis (TGA) was conducted using a Setaram thermal analyzer, model LabSys Evo. Samples were heated in an alumina crucible at a rate of 10 °C min^–1^ from 30 °C to 1000 °C under an inert atmosphere (N_2_). X-ray diffraction (XRD) was performed using a Rigaku diffractometer, model Miniflex 600, employing Cu Kα radiation (λ = 1.5406 Å) at 40 kV and 20 mA. Measurements were carried out over a 2θ range of 5° to 60° with a step size of 0.02° and a scan rate of 2° 2θ min^–1^. Peak indexing was performed using the databases of the International Zeolite Association (IZA) and the Crystallography Open Database (COD). Crystallinity was calculated according to eq 1,

1 $$\mathrm{CZ}\left(\%\right)=\frac{A_{t}}{A_{z}}\times 100$$

where CZ(%) is the percentage crystallinity of the zeolitic phase, and *A*_t_ and *A*_z_ are, respectively, the total area of the diffractogram and the area of the diffraction peaks corresponding to the obtained zeolitic phase.

Fourier-transform infrared spectroscopy (FTIR) was performed using an Agilent Cary 630 spectrometer. Spectra were obtained with powdered samples, with measurements carried out using 100 scan repetitions and a resolution of 2 cm^–1^. Scanning electron microscopy (SEM) was conducted using a JEOL JSM6610LV microscope, with an acceleration voltage adjustable between 300 V and 30 kV, resolution ranging from 3.0 to 15 nm, using a tungsten filament, and magnification from 5× to 300,000×. An energy-dispersive X-ray spectroscopy (EDS) detector from Bruker, model Xflash Detector 6|10, was coupled to the microscope, featuring an analysis area of 10 mm^2^ and an energy resolution of 121 eV for Mn Kα, 38 eV for C Kα, and 47 eV for F Kα (100,000 cps).

For inductively coupled plasma optical emission spectrometry (ICP-OES) analysis, an Optima 7000DV instrument (PerkinElmer, USA) was used. Method calibration was performed by external standardization in 2% v/v nitric acid. The operational conditions were: radiofrequency power of 1300 W, plasma gas flow of 15 L min^–1^, nebulizer gas flow of 0.8 L min^–1^, auxiliary gas flow of 0.8 L min^–1^, and sample aspiration rate of 1.5 mL min^–1^. Method accuracy was evaluated through recovery assays for the elements Al, K, Na, and Si using 2 mg L^–1^ fortifications.

Solid-state nuclear magnetic resonance (NMR) experiments were performed at room temperature using a Varian–Agilent 400 MHz spectrometer operating at a magnetic field of 9.4 T. The ^27^Al and ^29^Si resonance frequencies were 104.16 and 79.41 MHz, respectively. The ^27^Al NMR spectra were acquired using a 1 μs pulse, a repetition time of 1 s, and 800 accumulated transients, with a spectral width of 100 kHz and an acquisition time of 20.48 ms. The spectra were referenced to a 1.2 mol L^–1^ aqueous aluminum nitrate solution. The ^29^Si NMR spectra were recorded using a π/2 excitation pulse of 5.25 μs, a repetition time of 30 s, and an acquisition time of 32 ms.

## Results and Discussion

### Characterization of Ornamental Rock Waste and Zeolite Synthesis

The thermogravimetric analysis of OR (Figure 1) revealed an atypical behavior, of a slight mass gain up to 500 °C, possibly associated with structural reorganizations. In addition, the material exhibits high thermal stability, a characteristic favorable for applications requiring resistance to heating, but which, on the other hand, suggests low reactivity for zeolite synthesis without additional chemical modifications.

**Figure 1:**
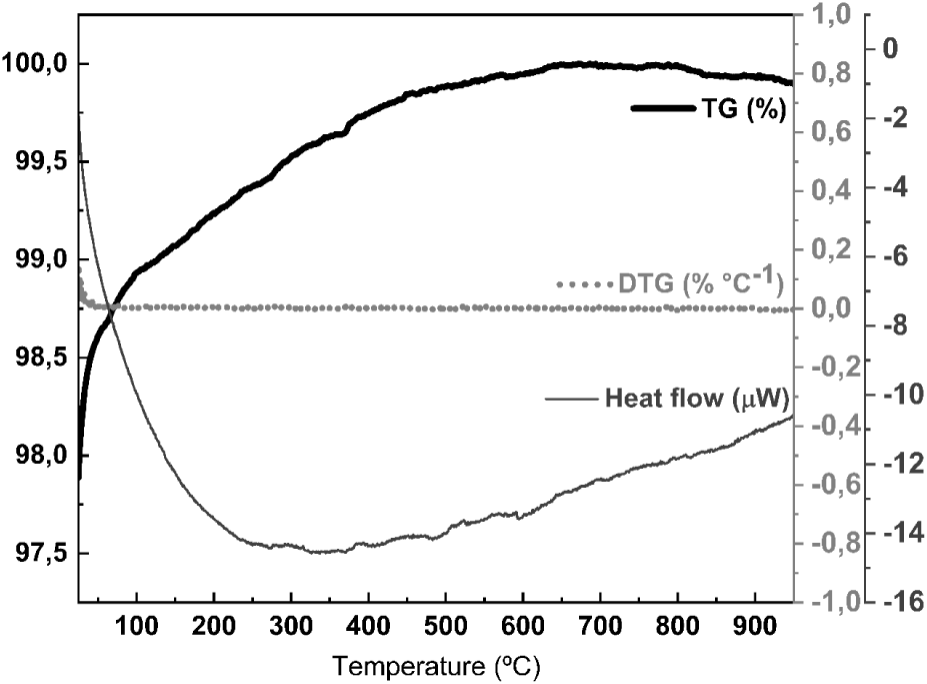
TGA analysis of ornamental rock waste.

The X-ray diffraction pattern of OR (Figure 2) confirmed the predominant presence of quartz and potassium feldspars (albite and orthoclase), crystalline phases with high thermal stability, which justifies the thermal behavior observed in the TGA. After calcination at 850 °C (CR850), the crystalline pattern was preserved, demonstrating that heating under this condition did not promote amorphization or significant structural changes. In contrast, alkaline activation at the same temperature (CR850-Na) resulted in complete amorphization of the material, evidenced by the absence of crystalline peaks and the appearance of a diffuse halo around 2θ ≈ 30°. This behavior confirms that alkaline fusion with NaOH was effective in disrupting the crystalline network of silicates and feldspars, producing a highly reactive amorphous material, an essential condition for its use as a precursor in zeolite synthesis.^35^

**Figure 2:**
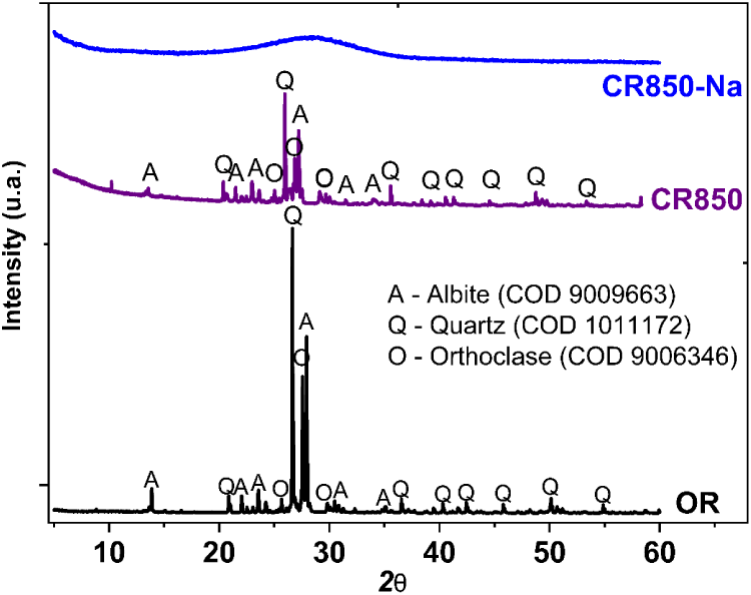
XRD analysis of the ornamental rock waste and products after waste treatment.

SEM/EDS analyses (Figure 3) showed that OR exhibited a relatively homogeneous distribution of silicon and aluminum within its crystalline matrix, consistent with the presence of quartz and feldspars identified by XRD. In contrast, CR850-Na displayed an irregular, fragmented, and porous morphology with vitreous regions, along with a homogeneous redistribution of the main elements throughout the material. The clear detection of sodium in CR850-Na confirms its incorporation during the alkaline activation process, resulting in a greater accessible surface area and increased chemical reactivity, which make this material a suitable precursor for zeolite synthesis.

**Figure 3:**
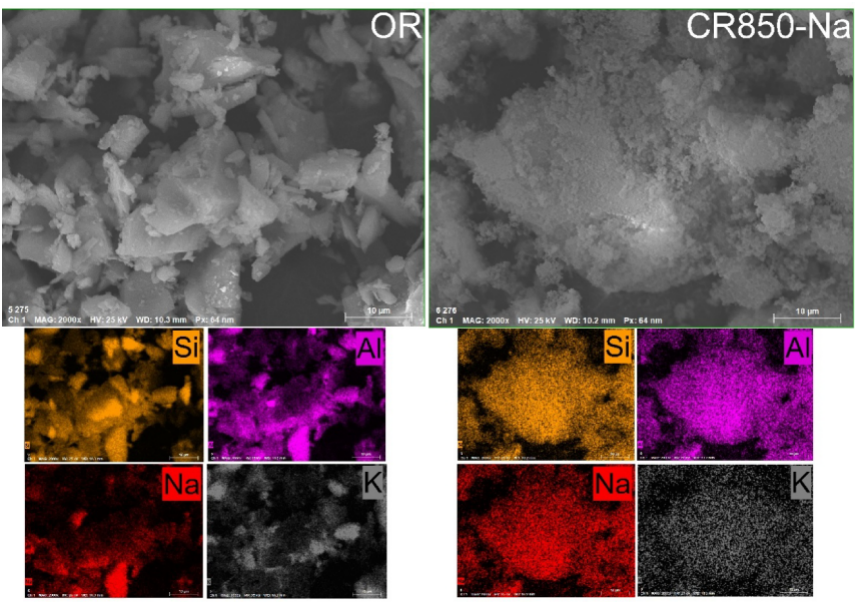
SEM/EDS analyses of the ornamental rock waste and products after waste treatment.

Kumar et al.^41^ reported the formation of amorphous precursors in the form of worm-like particles, with branched morphology, heterogeneous silica-rich composition, and metastable character, acting as nutrient reservoirs for nucleation and crystal growth. Thus, the characteristics of CR850-Na reinforce that this material may play an analogous role, functioning as a highly reactive intermediate phase, suitable for conversion into zeolitic structures under hydrothermal conditions.

The EDS analysis of CR850-Na (Figure 4A) revealed chemical changes compared to the original residue (OR). The OR exhibited a SiO_2_/Al_2_O_3_ mass ratio of 2.01, characteristic of silica-rich materials. After the steps of alkaline activation, dissolution, washing, and drying, this ratio decreased to 1.03, indicating the selective leaching of silicon and, consequently, the relative enrichment of aluminum in the solid matrix. The composition of the aqueous phase (LS-CM), resulting from the washing step of CR850-Na, was evaluated by ICP-OES (Figure 4B), confirming the leaching of silicon. The solution exhibited a silicon concentration approximately 15 times higher than that of aluminum, in addition to elevated sodium levels. This discrepancy explains the decrease in the SiO_2_/Al_2_O_3_ ratio in the residual solid, while also revealing the potential use of the liquid phase as an additional source of silicate precursors.

**Figure 4:**
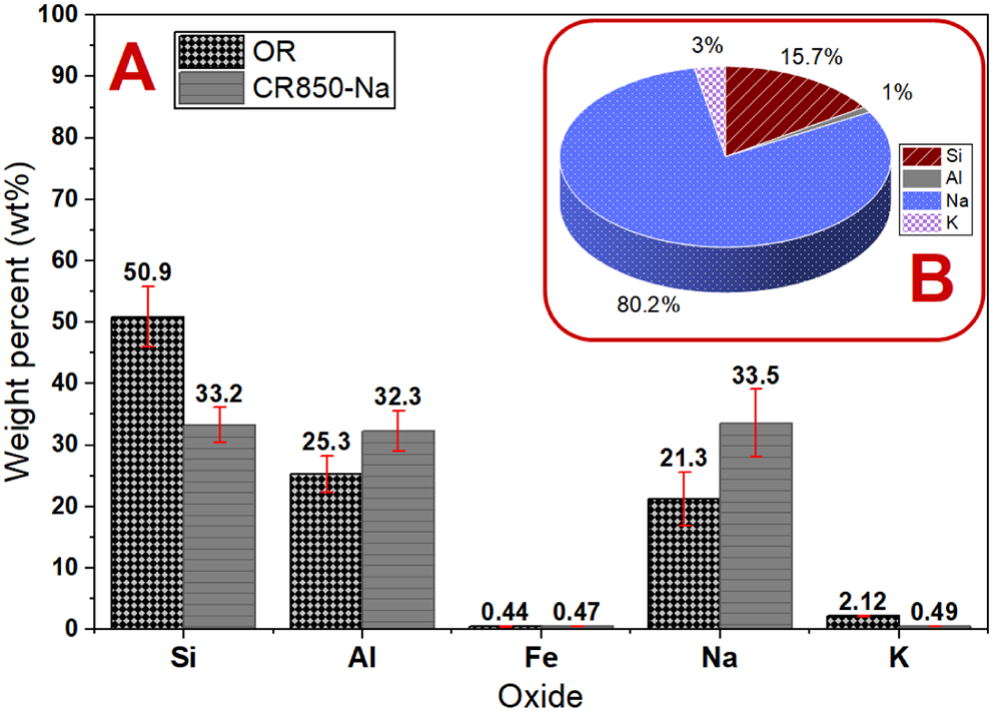
EDS (A) and ICP-OES (B) analyses of the ornamental rock waste and products after waste treatment.

Sodium incorporation into the CR850-Na was observed, originating from the alkaline activation, which represents a strategic factor for zeolite synthesis. The presence of Na^+^ cations is essential not only because they actively participate in nucleation and crystal growth processes, but also because they play a direct role in stabilizing the aluminosilicate framework. These ions compensate for the negative charges introduced by the isomorphic substitution of Al^3+^ in the lattice, thereby reducing structural mobility and preventing dealumination processes.^15^

Another relevant aspect is the influence of Na^+^ on acidity modulation: by occupying sites associated with Si–O–Al bridging oxygens, the cations decrease the density of Brønsted acid sites, making the structure more stable under severe reaction conditions while enhancing both thermal and hydrothermal stability of the zeolite.^42^ Thus, sodium incorporation into the precursor not only directs the formation of more stable crystalline nuclei but also provides decisive structural and chemical advantages for zeolite synthesis and performance.

Regarding the silicon-leached phase (LS-CM), it can also be employed for zeolite synthesis. Similar strategies have been reported in the literature, as described by Ndlovu et al.,^43^ in which the enrichment of the aqueous phase with silicon was exploited for the synthesis of zeolites with high Si/Al ratios, such as MFI- and GIS-type zeolites. Therefore, both the aluminosilicate solid and the resulting aqueous phase can be synergistically utilized for zeolite synthesis, enhancing process efficiency and reducing waste generation.

The presence of Fe in CR850-Na is a relevant factor for interpreting the structural evolution during zeolite synthesis. Although systematic studies addressing the direct effect of metallic impurities on LTA and sodalite zeolites are still limited, recent evidence indicates that species such as Fe, Ca, and Mg can influence nucleation, crystal growth, and phase transformation processes, especially in systems derived from waste materials or under highly alkaline conditions.^44–46^

Fe^3+^ ions may partially substitute Al^3+^ in the aluminosilicate framework, forming Fe–O–Si/Al linkages, which alters the local structural order, the effective Si/Al ratio, and the crystallinity. Similarly, divalent cations can disturb the charge balance and promote the formation of secondary phases, reducing LTA purity and accelerating its transformation into sodalite, a structurally more compact and thermodynamically stable phase in highly alkaline media.^45,47^

In the case of LTA zeolites, the Al content and its spatial distribution are crucial for maintaining the geometry of the supercages, and the presence of iron or other cations with variable coordination may introduce local distortions and reduce structural stability. In contrast, sodalite, whose structure is more compact and rigid, exhibits lower tolerance to cationic substitutions; thus, excess impurities tend to result in a higher amorphous fraction, smaller crystals, and a higher density of structural defects, with a direct impact on material quality and performance.^45^

From a functional perspective, the incorporation of Fe and other cations may block pores, reduce ion-exchange capacity, and compromise the thermal and hydrothermal stability of the material, with effects being more pronounced for LTA than for sodalite.^47,48^

### Characterization of the Zeolites

FTIR analysis revealed that the precursor material for synthesis was rich in hydrated oxides, evidenced by the presence of vibrations corresponding to O–H bond stretching at 3364 cm^–1^ and bending of adsorbed water at 1641 cm^–1^ (Figure 5). Furthermore, all generated products exhibited vibration signals related to aluminosilicates, with O–Si stretching at 981 cm^–1^ and Al–O stretching at 424 cm^–1^.^49^ It was observed that, after 12 h of crystallization, a band appeared at 555 cm^–1^, attributed to α-cage vibrations, characteristic of LTA-type zeolites. However, this band decreased in intensity after 24 h of crystallization, giving rise to bands at 657 cm^–1^, 698 cm^–1^, and 719 cm^–1^, which correspond to symmetric stretching modes of β-cages, characteristic of SOD-type zeolites,^49–51^ suggesting a possible phase transformation during the crystallization process.

**Figure 5:**
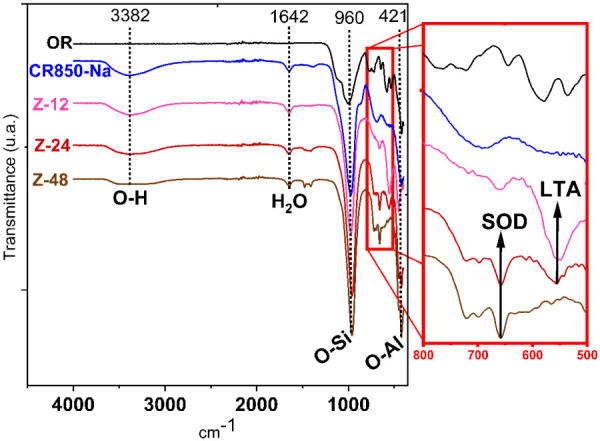
FTIR analyses of the zeolites obtained at different crystallization times and of the starting materials.

The phase transition was confirmed by XRD analysis (Figure 6) of samples collected at intervals of 6, 12, 24, and 48 h. It was observed that, after only 6 h of crystallization, LTA-type zeolite was formed as the sole phase, remaining so until 12 h. After 24 h, the coexistence of LTA and SOD phases was detected. Finally, after 48 h, SOD-type zeolite became the predominant phase, confirming the transition from LTA to SOD throughout the process.

**Figure 6:**
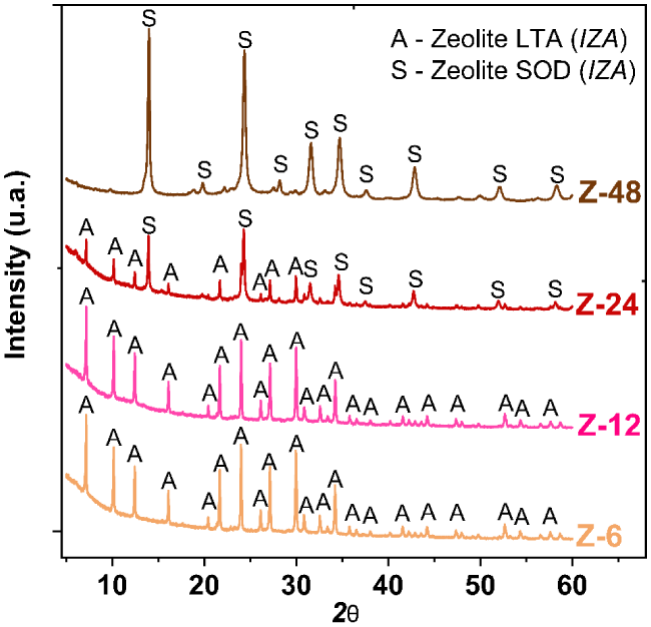
XRD analyses of the zeolites obtained at different crystallization times.

After 6 h of crystallization, the Z-6 sample corresponded to the LTA phase, as confirmed by the typical cubic morphology observed by SEM (Figure 7). After 12 h (Z-12), although LTA still predominated, signs of surface irregularity suggested the onset of a restructuring process, possibly associated with partial crystal dissolution. At 24 h (Z-24), the coexistence of remaining cubic particles and spherical crystals characteristic of sodalite indicated a transition stage, in which LTA began to be consumed by the nucleation and growth of the SOD phase. This process was completed after 48 h (Z-48), when the cubic crystals had almost completely disappeared, giving way to interconnected spherical aggregates typical of sodalite.

**Figure 7:**
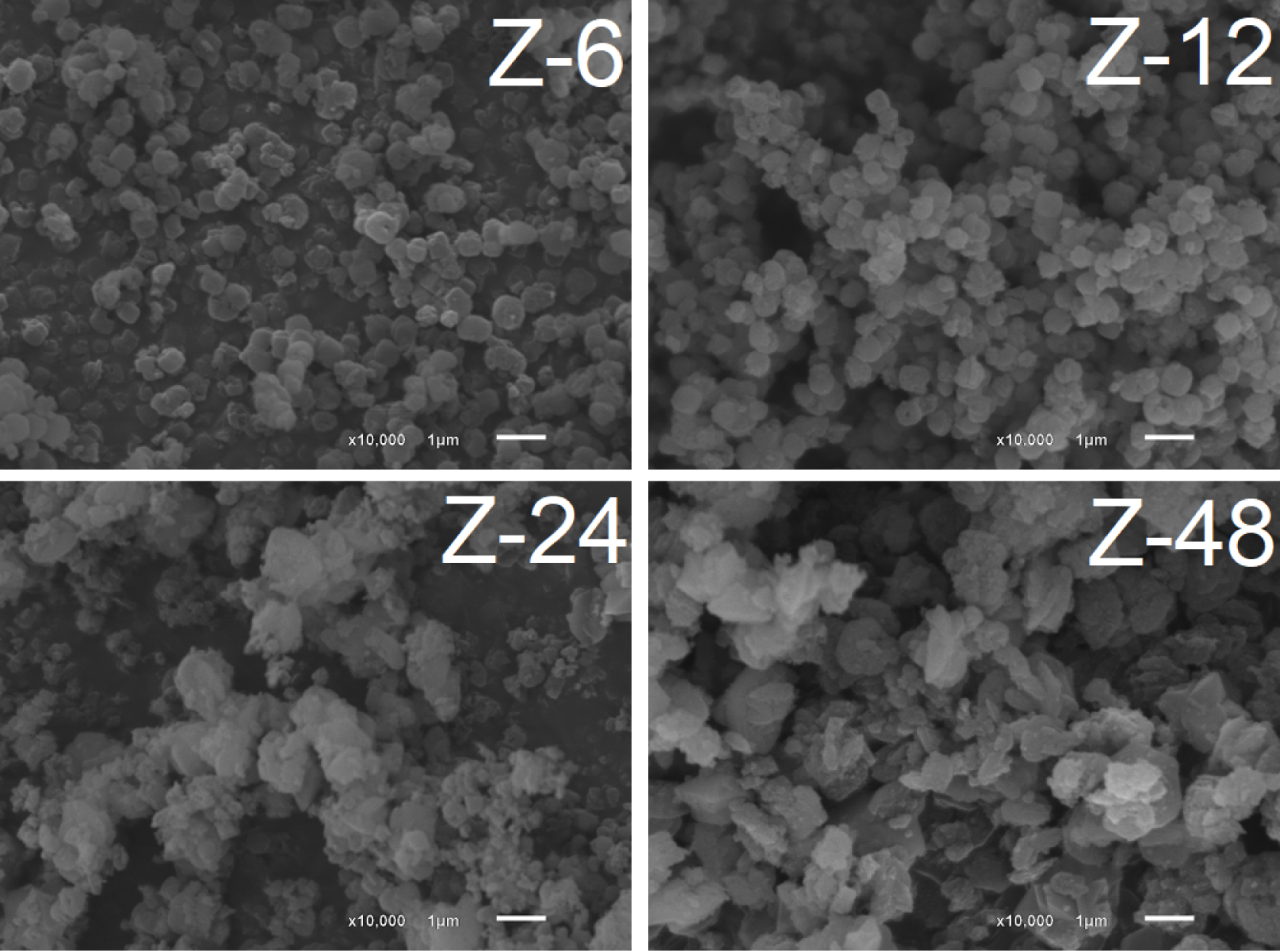
SEM analyses of the zeolites obtained at different crystallization times.

The results obtained in this study are consistent with the findings reported by Deng et al.,^42^ who demonstrated that increasing the hydrothermal temperature and prolonging the crystallization time favor the transformation of LTA zeolite into SOD, achieving complete conversion under more severe conditions (160 °C for 12 h). According to these authors, the dominant mechanism involves partial dissolution of the LTA phase followed by recrystallization into SOD, characterizing an Ostwald ripening-type process. This mechanism adequately explains the morphological features observed in the present work, such as the progressive corrosion of the cubic LTA surfaces, the formation of structural defects, and the subsequent emergence of spherical particles attributed to the SOD phase.

In a complementary manner, Peng et al.^52^ showed that the presence of specific anions, such as sulfate and carbonate, can accelerate this transformation by modulating the dissolution kinetics of LTA and the nucleation of SOD. These authors also reported preferential SOD growth at the edges and structurally less stable regions of LTA crystals, which corroborates the observations of this study, in which the transformation occurred gradually, initiating from the surface degradation of the cubic particles.

However, the literature indicates that the LTA → SOD transformation does not occur in a unique or universal manner, being strongly dependent on chemical conditions and crystallization history. Ding et al.^53^ and Djozing et al.^54^ demonstrated that under high alkalinity and different thermal regimes, the transformation may proceed through distinct mechanisms, including solid–solid restructuring and hybrid dissolution–recrystallization processes. Additional microstructural evidence, such as the formation of internal nanoplates and the progressive rupture of the cubic LTA “shell” described in the Greer et al.^55^ study, further supports that the transformation is governed not only by thermodynamic factors but also by crystal growth rates, localized nucleation, and defect evolution.

Moreover, studies based on natural raw materials, such as clays, show that LTA frequently forms as an initial metastable phase, evolving into SOD or multiphase systems as hydrothermal conditions become more severe. From an applied perspective, Ritter et al.^56^ highlighted that the choice of reagents and the control of alkalinity, crystallization time, and temperature are decisive for the final zeolitic phase obtained, reinforcing the need for an integrated mechanistic analysis.

The evolution of the Na_2_O/Al_2_O_3_ ratio throughout the crystallization process (Figure 8) provides additional evidence of the phase transformation from LTA to SOD. At 6 h (Z-6), the relatively low Na_2_O/Al_2_O_3_ value (0.60) is associated with the predominance of the highly crystalline LTA phase, consistent with a structure stabilized by a lower amount of compensating cations. Ritter et al.^56^ state that an increase in Na^+^ concentration favors the formation of sodalite zeolites. At 12 h (Z-12), the Na_2_O/Al_2_O_3_ ratio increased significantly to 1.37, coinciding with the reduction in LTA crystallinity and the onset of morphological irregularities, suggesting that the excess Na^+^ destabilized the structural units of LTA and favored SOD nucleation. This transitional behavior was confirmed at 24 h (Z-24), when the Na_2_O/Al_2_O_3_ ratio decreased to 0.84 and the coexistence of both phases was observed. Finally, at 48 h (Z-48), the ratio increased again (1.27), in agreement with the predominance of the SOD phase.

**Figure 8:**
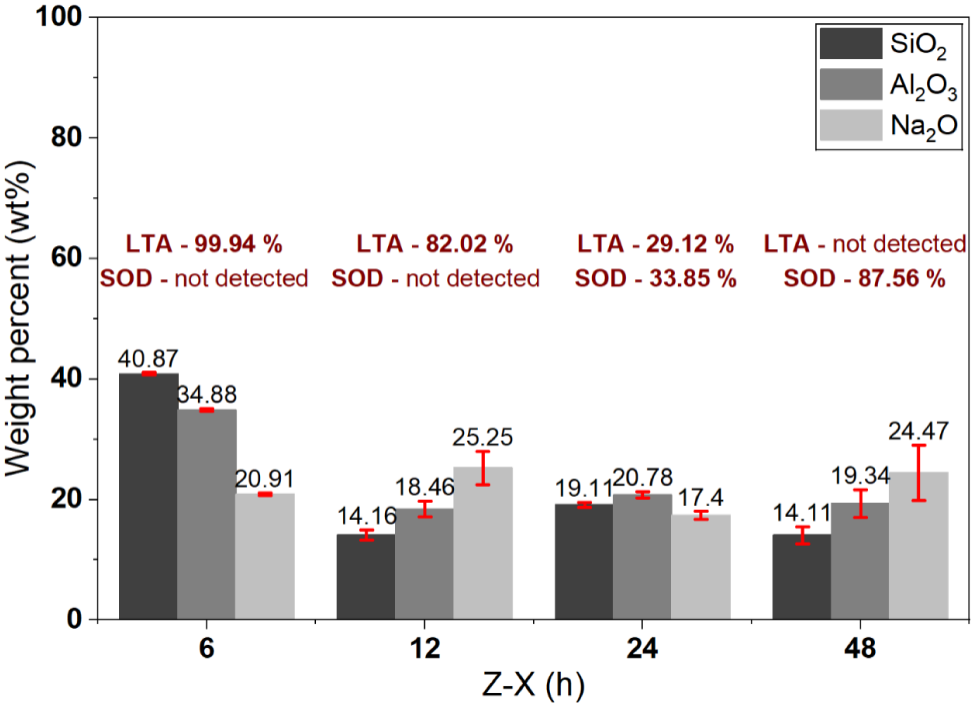
EDS analyses of the zeolites obtained at different crystallization times, along with the percentage crystallinity of each phase formed.

These results indicate that the higher Na_2_O/Al_2_O_3_ ratio acted as a determining factor for the formation of the SOD phase at the expense of LTA, demonstrating that the redistribution of sodium over the crystallization time, associated with the temporal evolution of the system, constituted the main driving force for the phase transition. Additional factors, such as temperature, alkalinity, and the nature of the anions, may also play a modulatory role in the process.

It is noteworthy that, in the present work, the crystallization of the zeolitic structures was conducted in a system without hydrothermal reactors, which are traditionally employed in syntheses of this type. Despite this methodological difference, the observed behavior was remarkably like that described in the literature, with the progressive transformation of LTA into SOD following the same temporal and morphological trend. These findings demonstrate that phase conversion can be achieved under simpler experimental conditions, suggesting that the use of hydrothermal reactors, although widely applied, is not strictly necessary to enable the transformation of LTA into SOD.

The comparative thermal analysis of materials Z-6 (LTA) and Z-48 (SOD) (Figure 9) reveals distinct behaviors related to thermal stability and mass loss processes. For both solids, an initial mass loss stage below 150 °C is observed, attributed to the removal of physically adsorbed water and water weakly retained within the channels and cavities of the zeolites. This process is accompanied by endothermic peaks in the DSC curve, typical of dehydration.

**Figure 9:**
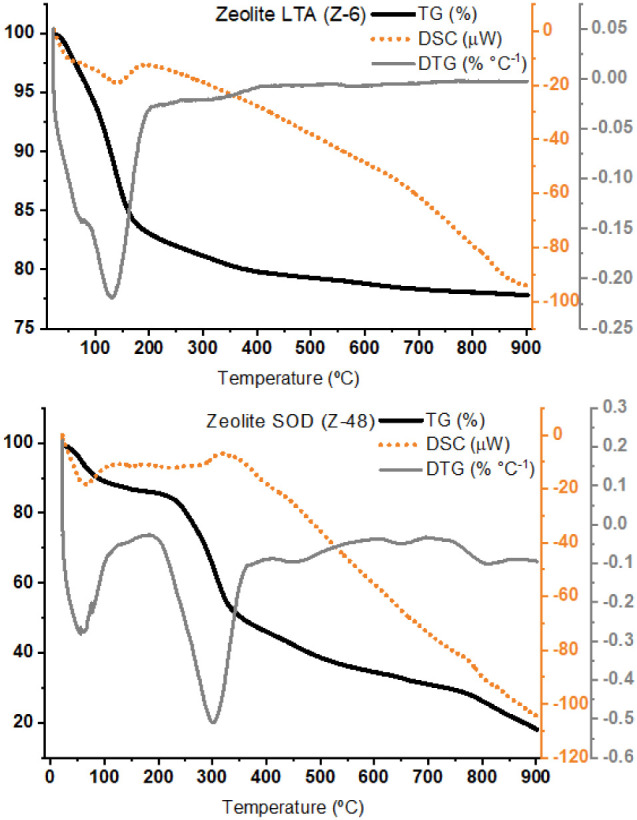
Thermal analysis of the obtained LTA (Z-6) and SOD (Z-48) zeolites.

In the case of the LTA zeolite (Z-6), after the initial dehydration stage, the TG curve remains relatively stable up to approximately 700 °C, indicating higher thermal resistance. Small variations in the DTG curve within this temperature range may be associated with the gradual removal of more strongly coordinated or trapped water molecules within the structure. Above 700 °C, a slight mass loss begins, accompanied by changes in the DSC baseline, suggesting deeper structural transformations, possibly related to disordering and the onset of partial collapse of the crystalline framework.

In contrast, the SOD zeolite (Z-48) exhibits a distinct behavior, with more pronounced mass losses occurring in two main stages: the first, between 50 and 200 °C, related to initial dehydration, and a second, more pronounced stage between 200 and 400 °C, which may be associated with the release of strongly retained water molecules and the onset of structural rearrangements. The thermal stability of this material is lower than that of LTA, as evidenced by the continued mass loss throughout the heating range up to 900 °C. Furthermore, the DSC curve exhibits more pronounced variations, including exothermic events around 800 °C, suggesting recrystallization processes or phase transformation.

Thus, the comparison between the two materials shows that the LTA zeolite (Z-6) exhibits greater thermal stability, maintaining its structure relatively preserved up to higher temperatures, whereas the SOD zeolite (Z-48) demonstrates less stable behavior, with more intense mass loss events and evidence of structural transformation at intermediate and high temperatures.

The LTA zeolite (Z-6) exhibited a thermal behavior quite similar to that reported by Ritter et al.,^56^ with dehydration events below 250 °C and high stability up to temperatures close to 700–800 °C, consistent with the profiles described for analogous materials synthesized under autoclave conditions. This result suggests that the absence of the autoclave-assisted crystallization step did not significantly compromise the thermal stability of LTA. In contrast, the SOD zeolite displayed more intense and continuous mass losses throughout heating, differing from the higher stability reported by the authors.

This difference may be related to the lower crystallinity or the higher degree of structural defects introduced by the employed synthesis route, factors that weaken the framework and anticipate thermal degradation. Therefore, while LTA maintained performance comparable to that described in the literature, SOD proved to be more sensitive to the absence of autoclave treatment, indicating that this condition differently affects the stability of the zeolitic phases formed.

The ^29^Si NMR spectra (Figure 10) indicate that the synthesized zeolites exhibit a predominantly condensed aluminosilicate framework, with resonances concentrated in the characteristic range of Q^4^(nAl) sites (≈ −85 to −95 ppm), which is typical of low-Si/Al zeolites.^57^ In sample Z6, a strong and well-defined resonance at approximately −85 ppm is observed, accompanied by a broader secondary contribution near −92 ppm. This spectral pattern is characteristic of highly aluminated Q^4^ environments, predominantly Q^4^(3Al) and Q^4^(2Al) species,^58^ in agreement with the high crystallinity of the LTA phase identified by XRD and with the low Na_2_O/Al_2_O_3_ ratio determined by EDS.

**Figure 10:**
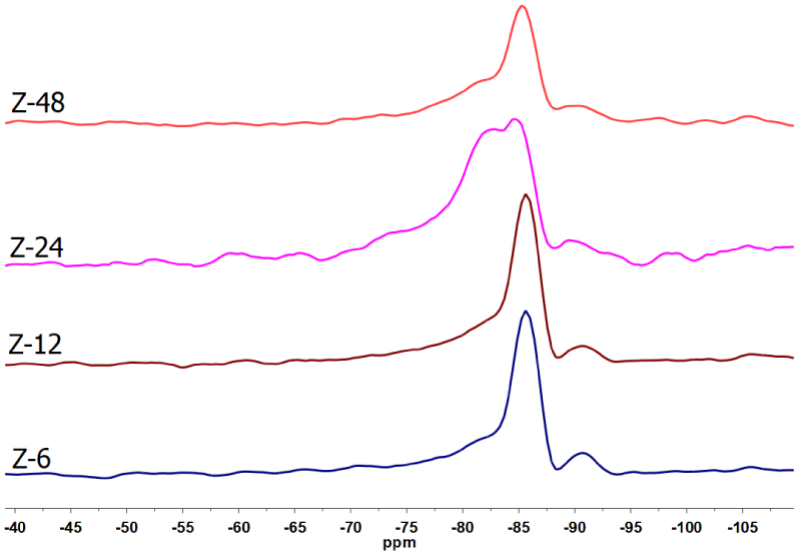
^29^Si NMR spectra of the obtained zeolites.

For Z12, the persistence of these resonances indicates that, despite the increase in Na^+^ content in the system, the local Si–O–Al connectivity remains largely preserved. This behavior is consistent with the partial reduction in LTA crystallinity observed by XRD and with the onset of morphological irregularities evidenced by SEM.

In sample Z24, the broadening of the resonance centered at −85 ppm, together with the emergence of a spectral contribution shifted toward less negative values around −83 ppm, reflects an increased heterogeneity of the Q^4^(nAl) environments. This evolution is directly associated with the coexistence of LTA and SOD phases identified by XRD and correlates with the redistribution of Na^+^ cations, as evidenced by the variation in the Na_2_O/Al_2_O_3_ ratio, characterizing an intermediate stage of structural reorganization.

Finally, in Z48, the attenuation of the contribution near −83 ppm and the reestablishment of a dominant resonance at −85 ppm indicate the stabilization of Q^4^(3Al) environments typical of sodalite, in full agreement with the predominance of the SOD phase observed by XRD. These results demonstrate that the LTA → SOD transformation is mainly governed by Na^+^ redistribution and local topological rearrangements of the aluminosilicate framework, as reported for low-silica zeolites.^57–59^ Thus, the ^29^Si NMR data consistently corroborate the structural evolution inferred from XRD, EDS, and Na_2_O/Al_2_O_3_ analyses, reinforcing that the transition from LTA to SOD is predominantly controlled by local framework rearrangements induced by the redistribution of compensating cations.

The ^27^Al NMR analysis of the zeolites revealed only a signal around 58 ppm (Figure 11), attributed to aluminum in tetrahedral coordination (AlO_4_), in which each aluminum atom is bonded to four silicon atoms via oxygen bridges. This result indicates a highly organized structure, without the presence of extraframework aluminum species in pentahedral or octahedral coordination, which could contribute to the material’s acidity.^60^

**Figure 11:**
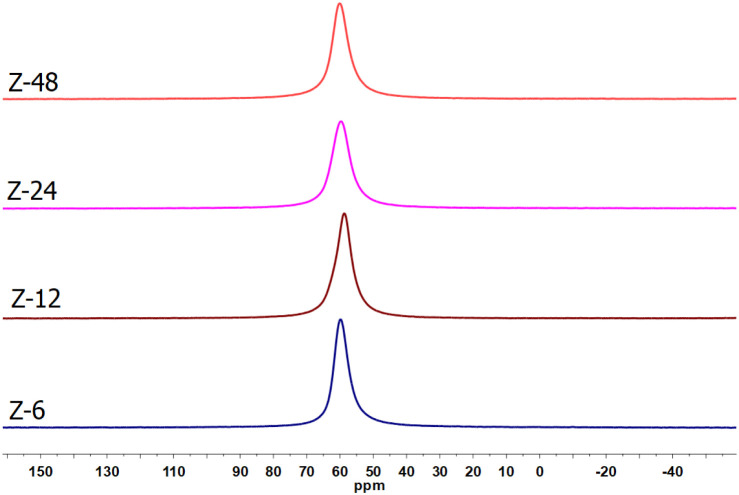
^27^Al NMR spectra of the obtained zeolites.

The structural organization of zeolites is closely associated with the Si/Al ratio. According to Xu et al.,^61^ an increase in this ratio in LTA-type zeolites promotes the formation of different aluminum species, identified by ^27^Al NMR: hexacoordinated aluminum (signal between −7.5 and 1.3 ppm), distorted tetrahedral aluminum (46.0 to 31.0 ppm), and pentacoordinated aluminum (31.0 to 17.1 ppm). These signals reflect not only the degree of structural organization of the zeolite but are also related to the presence of acidity within the crystalline framework.

In the present study, the composition of the obtained zeolites, in the sodium form due to the presence of Na^+^ cations from the synthesis medium acting as charge balancers, indicates the absence of Brønsted acid sites, which arise when the compensating cations are H^+^. Furthermore, the lack of octahedrally coordinated aluminum species, which, after thermal treatment of hydrated zeolites, generate Lewis acid sites, also suggests the absence of Lewis acidity.^62^ Thus, both the SOD and LTA zeolites obtained in this work exhibit a basic character, making them promising for reactions involving the deprotonation of acidic hydrogens, acting as catalysts in organic processes.^63^

LTA zeolites have demonstrated efficiency in the removal of hazardous inorganic and organic substances from water.^64^ Their production from waste materials and the short crystallization time can be strategies to reduce production costs. Moreover, the use of waste in the synthesis of new materials is a practice that deserves encouragement, as it aligns with the principles of sustainable chemistry. Finally, the additional benefit of being able to perform the synthesis without the need for hydrothermal reactors broadens the feasibility of using these materials on a large scale.

## Conclusion

This study demonstrated that ornamental rock wastes rich in quartz and potassium feldspar can be efficiently converted into precursors for zeolite synthesis via alkaline activation, adding value to industrial residues and promoting a sustainable production route. Alkaline activation at 850 °C resulted in complete amorphization of the material, a reduction in the Si/Al ratio from 2.01 to 1.03, indicating selective silicon leaching, and sodium incorporation, which is essential for zeolite synthesis.

Nonautoclave-assisted hydrothermal crystallization enabled the initial formation of LTA zeolite (Z-6) with 99.94% crystallinity and characteristic cubic morphology in just 6 h, followed by a progressive transition to SOD zeolite (Z-48), reaching 87.56% crystallinity after 48 h. Sodium redistribution, evidenced by variations in the Na_2_O/Al_2_O_3_ ratio (0.60 in Z-6 → 1.37 in Z-12 → 0.84 in Z-24 → 1.27 in Z-48), was a key factor driving the phase transformation.

^29^Si and ^27^Al NMR analyses confirmed the structural evolution: ^29^Si revealed local rearrangements of the aluminosilicate framework associated with Na^+^ redistribution during the LTA → SOD transition, while ^27^Al showed the exclusive presence of tetrahedral aluminum (∼58 ppm), indicating the absence of extraframework species and Brønsted or Lewis acid sites, thus conferring a basic character to the zeolites. Thermal analyses revealed marked differences between the phases: LTA maintained stability up to ∼700 °C, whereas SOD exhibited more pronounced mass losses in two stages, along with structural changes and exothermic events near 800 °C.

Therefore, this work demonstrates that the synthesis of LTA and SOD zeolites from ornamental rock wastes is feasible without hydrothermal reactors, combining short crystallization times, waste valorization, and the production of materials with tunable structural and chemical properties, thereby expanding their potential for applications such as catalysts, adsorbents, sensors, and gas separation.
